# A Bioadhesive Barrier-Forming Oral Liquid Gel Improved Oral Mucositis and Nutritional Status in Patients With Head and Neck Cancers Undergoing Radiotherapy: A Retrospective Single Center Study

**DOI:** 10.3389/fonc.2021.617392

**Published:** 2021-02-22

**Authors:** Jinlong Wei, Jie Wu, Huanhuan Wang, Bin Wang, Tingting Zhao, Lingbin Meng, Lihua Dong, Xin Jiang

**Affiliations:** ^1^ Department of Radiation Oncology, The First Hospital, Jilin University, Changchun, China; ^2^ Jilin Provincial Key Laboratory of Radiation Oncology & Therapy, The First Hospital of Jilin University, Changchun, China; ^3^ NHC Key Laboratory of Radiobiology, School of Public Health, Jilin University, Changchun, China; ^4^ Department of Hematology and Medical Oncology, Moffitt Cancer Center, Tampa, FL, United States; ^5^ Key Laboratory of Pathobiology, Ministry of Education, Jilin University, Changchun, China

**Keywords:** Episil^®^, head and neck cancer, nutritional status, oral mucositis, radiotherapy

## Abstract

**Background:**

Episil^®^ is a bioadhesive barrier-forming oral liquid gel that can relieve oral mucositis (OM) caused by radiotherapy (RT) and hence relieves pain effectively. In this study, we observed the effects of Episil^®^ on the OM and nutritional status of patients with head and neck cancers (HNCs) undergoing RT.

**Methods:**

A total of 50 HNC patients were divided into the Episil^®^ (25 patients) and control (25 patients) groups. Patients in the Episil^®^ group were sprayed with Episil^®^. In the control group, the kangfuxin solution or Kangsu™ oral gargle was used. Medical staff assessed the OM extent and timing as well as the nutritional status during treatment and recorded adverse reactions other than OM. The nutritional status assessment included the following indicators: Patient Generated-Subjective Global Assessment (PG-SGA) score, body mass index (BMI), body weight, albumin levels, and other hematological indicators.

**Results:**

The incidence of high-level OM (III–IV) after RT was lower in the Episil^®^ group than in the control group (*P* < 0.05). Nutritional status assessments showed that the Episil^®^ group had a lower percentage of weight loss than the control group at weeks 4 and 7 after RT. Similar results were also obtained for BMI and albumin levels (*P* < 0.05). Moreover, according to PG-SGA scores, fewer patients in the Episil^®^ group were malnourished and more patients were well-nourished (*P* < 0.05) compared with the control group.

**Conclusion:**

Episil^®^ effectively improved OM and malnutrition in HNC patients who received RT and has a good clinical application value.

## Introduction

Head and neck cancers (HNCs) are common tumor types whose main treatment option is radiotherapy (RT) (1–3). However, oral mucositis (OM) is a serious and common adverse reaction after RT. Since the RT target in HNCs includes the primary tumor site and the cervical metastatic lymph nodes, the oral mucosa becomes inevitably exposed to a certain dose of radiation, causing OM (4). The clinical symptoms of OM usually appear after the cumulative dose of approximately 15 Gy and reach a relatively severe degree at 30 Gy, lasting for several weeks or months (5). Moreover, OM can cause patients to have a dry mouth, difficulty in opening the mouth, and pain when swallowing, as well as other symptoms, resulting in reduced food intake and malnutrition, thus affecting the quality of life and the course of RT. A small number of patients even stop RT owing to severe OM symptoms, resulting in the delay of treatment time, which in turn affects overall treatment efficacy and patient survival (6, 7).

Malnutrition is very common during RT in patients with cancer. The treatment toxicity can lead to inadequate nutritional intake, which increases malnutrition risk (8, 9). In fact, the prevalence of malnutrition among HNC patients is estimated to be between 50% and 70% (10). In addition to pain when swallowing and dysphagia caused by a primary tumor, HNC radiation-induced OM may be the main cause. Severe radiation-induced OM can even make it difficult for patients to swallow a drop of water because of the pain (11). To date, however, there is still a lack of medication and treatment methods to relieve radiation-induced OM (12). In the face of such malnutrition in clinical practice, tube feeding, parenteral nutrition, and even gastrostomy are usually considered to prevent patients from having more serious consequences. However, these methods inevitably lead to increased hospitalization costs, increased complications, and worse (13, 14). Therefore, we hope to find appropriate medication and methods to alleviate the symptoms of OM and improve malnutrition among patients.

Episil^®^ is a bioadhesive barrier-forming oral liquid gel that can relieve the symptoms of OM caused by RT by effectively reducing pain (15, 16). Oral liquid gels are made up of lipids and preservative-free liquids and are kept in multi-dose containers. Upon contact with the oral mucosa, the fluid adheres and, within 5 min, forms a protective membrane that acts as a mechanical barrier to relieve pain. Episil^®^ contains soybean lecithin and diolein. Oil accumulates on the surface of the saliva and spontaneously forms a ball of shape. The spheres are connected to each other and quickly arranged into a thin gel skeleton, forming the physical barrier. The physical barrier has a strong biological adhesion. It sticks closely to the oral mucosa and spreads out to cover the oral mucosa to provide protection. A study showed that an average of 67.5% of the oral mucosa could be covered 3 h after administration (17). At present, this oral liquid gel has been clinically registered and approved as a medical device in the United States, the United Arab Emirates, Israel, and the European Union (18).

The main purpose of this clinical study was to evaluate the impact of Episil^®^ on RT-induced OM and the nutritional status of HNC patients. The results of this study may provide a better method for the treatment of OM caused by RT and the related malnutrition among patients.

## Methods

### Study Population

Data from 50 HNC patients treated in our center from 2018 to 2020 were retrospectively analyzed. Patients enrolled in the study were required to meet the following criteria: (1) the patient was diagnosed through histopathology as having HNC; (2) the patient was aged ≥18 years and could be either male or female; (3) the patient did not have serious endocrine and metabolic diseases; (4) the patient developed OM during RT; and (5) the patient had an Eastern Cooperative Oncology Group (ECOG) score ≤3 points. The exclusion criteria of this study were as follows: (1) Combined with severe chronic diseases; (2) Patients with mental illness and severe cognitive impairment; (3) Patients who refuse follow-up. A total of 50 HNC patients were segmented into the Episil^®^ group (25 patients) and the control group (25 patients).

### Study Treatment

All of the patients included in the study received RT. The RT technique involved volumetric arc intensity-modulated RT or intensity-modulated RT. The overall therapeutic irradiation dose was between 60 Gy and 74 Gy, and the RT was performed once a day, 5 times a week for 6-7 weeks. In addition, some of the patients in both groups received concurrent chemotherapy. The specific chemotherapy regimens included tegafur-gimeracil-oteracil-potassium (80 mg/m^2^/3 w) or cisplatin (30 mg/m^2^/w).

### Intervention for OM

All of the study participants underwent an oral examination by an oral surgeon prior to RT to determine if there were any abnormalities. OM was found in both groups after RT. Patients in the Episil^®^ group were administered with 1–3 sprays of the liquid at a frequency of 2–3 times per day to form a thin protective film that may act as a mechanical barrier in the oral cavity. In the control group, 10 ml kangfuxin solution or Kangsu™ oral gargle was gargled, 10 min at a time, 3 times per day. The therapeutic intervention time in the two groups was recorded from the beginning of RT to the disappearance of OM after RT. In addition, the patients from both groups had gargled with warm water before drug intervention to keep their oral cavity clean. During the occurrence of severe OM, hormones were used for a short period of time. At the same time, antibiotics or antifungal agents were also considered based on the patient’s sensitivity during the pharyngeal swab culture.

### Observed Indicators

#### OM

During the treatment, the patients’ OM was monitored daily. The time and degree of OM were recorded and evaluated according to the Radiation Therapy Oncology Group (RTOG) standard. The RTOG scoring criteria for radiation-induced OM were as follows: Level 0 – no change; Level I – hyperemia/mild pain, no painkillers needed; Level II – flaky mucositis or inflammatory serum and blood secretions or moderate pain, requiring pain medication; Level III – fused fibrous mucositis/severe pain, requiring anesthetics; Level IV – ulcer, hemorrhage, or necrosis (4). High-level (III/IV) OM served as an important indicator for our observations.

#### Pain Relief

We assessed pain relief after initial drug intervention in the Episil^®^ and control groups. To minimize the impact of confounding factors, we discontinued all pain medications including opioids 24 h before evaluating pain relief indicators. All patients were rated for oral mucosa pain at various time points (30 min, 1 h, 2 h, 4 h, and 6 h) as baseline and within 6 h of initial drug intervention. Pain in the oral mucosa was assessed using a numerical score (0–10 in the Likert scale).

#### Nutritional Status

Measures of nutritional status included weight, body mass index (BMI), hemoglobin, total lymphocyte count, albumin, prealbumin, and Patient Generated–Subjective Global Assessment (PG-SGA) score (19). The body weight of the patients during hospitalization was measured using a height and weight instrument (TCS-200-RT, China). The patients wore hospital gowns and were measured on empty stomachs after defecation. Blood indexes were evaluated using regular blood routine and biochemical tests. PG-SGA is a subjective assessment of the patient’s overall nutritional status (20–22). It was regularly evaluated by the medical staff at our center through a specific questionnaire. Each patient was divided into three levels based on the PG-SGA score, including severe malnutrition (PG-SGA C), moderate malnutrition (PG-SGA B), and good nourishment (PG-SGA A).

#### Other Adverse Reactions

Other adverse reactions, except for RT-induced OM, including xerostomia, nausea, vomiting, thrombocytopenia, neutropenia, neurotoxicity, and nephrotoxicity, were also recorded during the treatment.

### Ethics Approval and Consent to Participate

This study was approved by the Ethics Committee of the First Hospital of Jilin University. Informed consent was obtained from all patients who participated in the study. All studies were conducted in accordance with the relevant regulations and guidelines.

### Statistical Analyses

IBM SPSS version 24.0 was used for all statistical analyses. The chi-square test was used for count data. The measurement data were expressed as the mean ± standard deviation (SD) and were analyzed using the t-test. *P* < 0.05 indicated a statistically significant difference.

## Results

### Subject Characteristics

The baseline characteristics of the patients are shown in **Table 1**. Both groups of patients completed all of their treatments, and none discontinued treatment owing to exceptional circumstances. There were no significant differences observed in terms of age, weight, BMI, albumin, tumor type, or treatment between the two groups (*P* > 0.05).

**Table 1:** Baseline characteristics of Episil^®^ group and control group.

Characteristics	Episil^®^ group(n = 25)	Control group(n = 25)	*p* value
Age (years)	55.0 ± 12.8	54.8 ± 9.7	0.941
Sex ratio (M/F)	21/4	21/4	1.000
Weight (kg)	65.2 ± 10.7	66.8 ± 7.8	0.532
Body mass index (kg/m^2^)	22.5 ± 3.1	23.0 ± 2.2	0.532
Albumin (g/L)	38.2 ± 5.4	39.9 ± 2.7	0.173
Cancer type			
Nasopharyngeal carcinoma (%)	10 (40.0)	15 (60.0)	
Laryngeal cancer (%)	6 (24.0)	6 (24.0)	0.231
Oropharyngeal cancer (%)	9 (36.0)	4 (16.0)	
Therapy			
Radiotherapy (%)	12 (48.0)	10 (40.0)	0.569
Radiotherapy + chemotherapy (%)	13 (52.0)	15 (60.0)	

Continuous variables presented as mean ± SD. Categorical variables are presented as counts (%).

### OM

**Figure 1:**
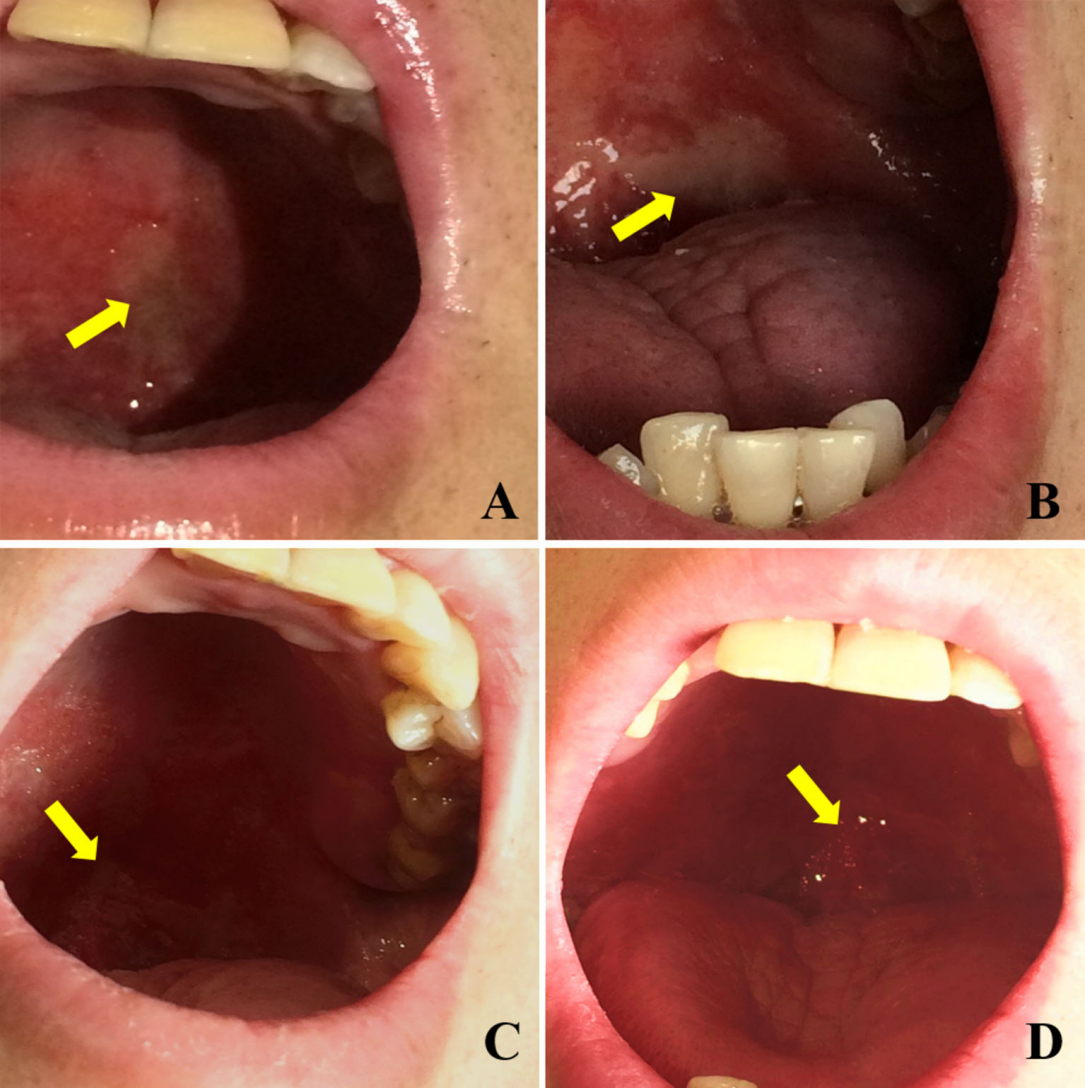
Relief of oral mucositis (OM) in patients with Episil^®^ group. **(A)** The OM after 20 times of radiotherapy; **(B)** The OM after 25 times of radiotherapy; **(C)** The OM after 29 times of radiotherapy; **(D)** The OM after 33 times of radiotherapy. Yellow arrows represent the surface of OM.

The relief of OM in patients within the Episil^®^ group is shown in **Figure 1**. A patient with a nasal tumor in the Episil^®^ group developed multiple 2-cm ulcers at the surface of the oral mucosa after 20 RT sessions. After 25 RT sessions, the small ulcers gradually fused into large ulcers. From then on, Episil^®^ continuously provided relief from OM. The ulcer surface gradually shrunk after 29 RT sessions, and the ulcer became close to remission after 33 RT sessions.

The OM results in the Episil^®^ and control groups are shown in **Table 2**. After RT, 5 and 12 patients in the Episil^®^ and control groups, respectively, developed high-level OM (III/IV). And the incidence of high-level OM (III–IV) after RT was lower in the Episil^®^ group than in the control group (*P* < 0.05).

**Table 2:** Result of oral mucositis in Episil^®^ group and control group.

	Low level oral mucositis (0、I、II)	High-level oral mucositis (III、IV)	χ_2_	*P* value
Episil^®^ group (n = 25)	20	5		
Control group (n = 25)	13	12	4.367	0.037*

*Statistical significance is reported at p < 0.05.

### Pain Relief

**Figure 2:**
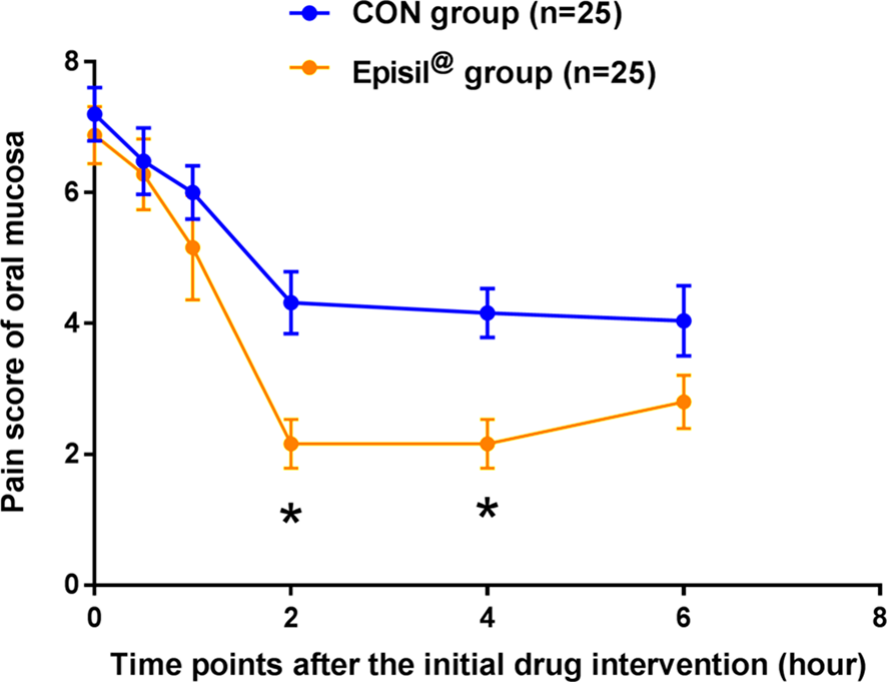
Pain score of oral mucosa at each time point after the initial drug intervention. Patients in both groups were rated for oral mucosa pain at each time point (30 min, 1 h, 2 h, 4 h, and 6 h) at baseline and within 6 h of initial drug intervention. Blue arrows represent the control group and orange arrows represent the Episil^®^ group. Data were expressed as the mean ± S.D (^*^ *P* < 0.05 *vs*. CON).

The oral mucosal pain in the Episil^®^ group and the control group at various time points and within 6 h of the first use of the drug is shown in **Figure 2**. The decrease in the intensity of oral mucosal pain at 2 and 4 h after using Episil^®^ compared to baseline was better than that of the control group (*P* < 0.05). There was no statistically significant difference between the Episil^®^ and the control groups in terms of the intensity of oral mucosal pain reduction 30 min, 1 h, and 6 h after the initial medication (*P* > 0.05). However, within 6 h of drug use the oral mucosal pain scores were significantly lower than the baseline in both groups. This indicates that Episil^®^ can significantly reduce oral mucosal pain after a single use, with the decrease in the oral mucosal pain intensity within 2–4 h being better than the baseline in the control group.

### Nutritional Status

**Figure 3:**
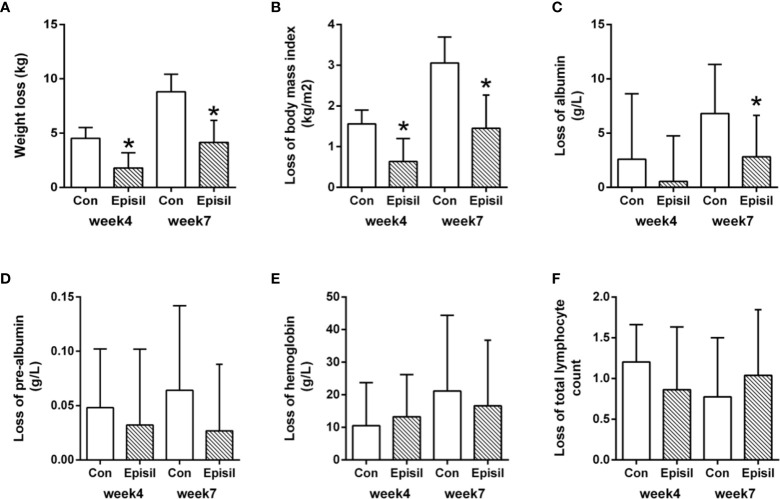
Nutritional Status of Episil^®^ group and control group. **(A)** The weight loss at week 4 and week 7; **(B)** The loss of body mass index at week 4 and week 7; **(C)** The loss of albumin at week 4 and week 7; **(D)** The loss of pre-albumin at week 4 and week 7; **(E)** The loss of hemoglobin at week 4 and week 7; **(F)** The loss of total lymphocyte count at week 4 and week 7. Data were expressed as the mean ± SD (*P < 0.05 vs. CON).

The nutritional status assessment results for the Episil^®^ and control groups are shown in **Figure 3** and **Table 3**. At 4 and 7 weeks after RT, the weight and BMI loss in the Episil^®^ group were more significant than those in the control group (*P* < 0.05). The reduction of albumin was more obvious in the control group than in the Episil^®^ group at 7 weeks after RT (*P* < 0.05), but at 4 weeks after RT, there was no statistical difference between the two groups (*P* > 0.05). At 4 and 7 weeks after RT, the pre-albumin level, hemoglobin, and total lymphocyte count index of the Episil^®^ and control groups decreased, but the difference was not statistically significant (*P* > 0.05).

**Table 3:** Nutritional Status of Episil^®^ group and control group.

Outcome		Episil^®^ groupMean ± SD	Control group. Mean ± SD	*P* value
Weight loss (kg)	Week4 Week7	1.80 ± 1.39 4.14 ± 2.01	4.52 ± 1.01 8.80 ± 1.63	0.000* 0.000*
Loss of body mass index (kg/m^2^)	Week4 Week7	0.64 ± 0.56 1.45 ± 0.82	1.56 ± 0.34 3.05 ± 0.64	0.000* 0.000*
Loss of albumin (g/L)	Week4 Week7	0.56 ± 4.20 2.82 ± 3.81	2.60 ± 6.03 6.80 ± 4.51	0.171 0.002*
Loss of pre-albumin (g/L)	Week4 Week7	0.03 ± 0.07 0.03 ± 0.06	0.05 ± 0.05 0.06 ± 0.08	0.371 0.067
Loss of hemoglobin (g/L)	Week4 Week7	13.2 ± 12.9 16.60 ± 20.10	10.50 ± 13.20 23.96 ± 29.86	0.458 0.312
Loss of total lymphocyte count	Week4 Week7	0.86 ± 0.77 1.04 ± 0.81	1.20 ± 0.46 0.77 ± 0.72	0.064 0.233

*Statistical significance is reported at p < 0.05.

The assessment results of the PG-SGA scores of the two groups are shown in **Table 4**. At 4 and 7 weeks after RT, more patients were assessed as well-nourished and fewer as malnourished in the Episil^®^ group than in the control group. However, only the difference in results at week 7 was statistically significant (*P* < 0.05).

**Table 4:** Nutritional status as defined by PG-SGA global rating for Episil^®^ group and control group.

Nutritional status	Week 0		Week 4		Week 7	
	Episil^®^	Con	Episil^®^	Con	Episil^®^	Con
Well nourished (PG-SGA A)	17	16	10	7	14	4
Malnourished (PG-SGA B, C)	8	9	15	18	11	21
*P* value^#^		0.765		0.370		0.003*

PG-SGA, Patient Generated-Subjective Global Assessment.

^#^Chi square analysis.

*Statistical significance is reported at p < 0.05.

### Other Adverse Reactions

The results of the adverse reaction assessment are shown in **Table 5**. There was no significant difference between the Episil^®^ group and the control group in terms of xerostomia, nausea, vomiting, thrombocytopenia, neutropenia, neurotoxicity, nephrotoxicity, and other adverse reactions (*P* > 0.05).

**Table 5:** Statistical results of other adverse reactions induced by radiotherapy for Episil^®^ group and control group.

	Episil^®^ group (n = 25)	Control Group (n = 25)	*p* value
Thrombocytopenia	4	2	0.663
Neutropenia	6	5	0.733
Nausea	7	8	0.758
Vomiting	3	5	0.699
Xerostomia	1	3	0.602
Nephrotoxicity	0	0	ns
Neurotoxicity	0	0	ns

Data are expressed as number of patients; ns, not significant.

## Discussion

The prevalence of malnutrition in patients receiving RT for HNCs is relatively high, with OM caused by RT probably being the main cause (23, 24). Patients with severe OM often have difficulty maintaining a normal diet and nutrition owing to the pain when swallowing (25). In this study, we found that Episil^®^ could relieve the OM caused by RT as well as the associated mucosal pain. In addition, the patients treated with Episil^®^ had a satisfactory nutritional status. These findings may have resulted from the relief of the patients’ oral mucosal pain, enabling them to receive good oral nutritional support during the treatment.

The prevention and treatment of radiation-induced OM has always been given attention (26, 27). Although there are many drugs and treatments, including growth factors and cytokines (28), anti-inflammatory medications (29), antimicrobial medication (30), natural medication (31), and cryotherapy (32), that can target OM clinically, their effects remain inconsistent. Moreover, most of these treatments lack evidence from controlled clinical trials, and their therapeutic effects are not obvious, indicating that OM is not yet completely solved (26). Wong et al. (33) studied the therapeutic and preventive effects of antibacterial rinsing using the Caphosol^®^ mouthwash on radiation-induced OM. The results showed that Caphosol^®^ users were less likely to develop grade IV OM, but these results were not statistically significant. By contrast, our study results showed that the incidence of high-level OM (III-IV) was lower in the Episil^®^ group than in the control group after RT *(P* < 0.05). After treatment with Episil^®^, the damaged mucosa was better protected and repaired, and the oral cavity improved; hence, the mucositis reaction became less severe.

OM may lead to severe oral mucosal pain in patients receiving RT for HNCs, requiring more enteral or parenteral nutrition, supportive care, opioid analgesics, and hospitalization (34). Moreover, patients who received large doses of opioid analgesics still experienced severe pain and difficulty in drinking and eating (35). However, Cheng et al. (18) conducted a multi-center randomized study showing that Episil^®^ displayed effective local analgesia for cancer patients with OM after chemotherapy and/or RT. Hadjieva et al. (15) have also shown that Episil^®^ is effective in alleviating pain in patients with OM associated with RT for HNC. Pain relief is immediate and noticeable and lasts up to 8 h. In our study, we found that oral mucosal pain in patients became significantly reduced after a single use of Episil^®^ and that the oral mucosal pain score within 6 h was lower than that at baseline. Moreover, the decrease in oral mucosal pain intensity at 2–4 h was significantly better than that in the control group (*P* < 0.05). Episil^®^ rapidly forms a protective membrane in the oral cavity that acts as a mechanical barrier, which may have been the key to oral mucosal pain relief.

When HNC patients receiving RT suffer from malnutrition due to limited food intake owing to OM, maintaining a good nutritional status through the use of conventional nutritional therapy, including enteral nutrition and parenteral nutrition, can be difficult (36, 37) because these treatments cannot entirely replace oral nutrition. Our results showed that nutritional status indicators among patients in the Episil^®^ group, including body weight, BMI, and albumin, were maintained better than those in the control group at 4 and 7 weeks after RT. Although there was no statistical difference in terms of the decrease of prealbumin, hemoglobin, total lymphocyte count, and other nutritional indicators between the two groups, these indicators demonstrated slightly better results in the Episil^®^ group than in the control group. In addition, PG-SGA score results showed that at weeks 4 and 7 after RT, fewer patients in the Episil^®^ group were assessed as malnourished and more as well-nourished compared with the control group. These findings indicate that the nutritional status of patients improved after treatment with the oral mucosa protectant Episil^®^. Therefore, relieving OM and oral mucosal pain may be key factors in improving the eating and nutritional status of HNC patients receiving RT.

The limitation of our study is its retrospective nature and small sample size. Future clinical studies should accumulate more data, and prospective analyses should be conducted.

In conclusion, Episil^®^ as a bioadhesive barrier-forming oral liquid gel can effectively improve OM and malnutrition in patients with HNCs undergoing RT and therefore has a good clinical application value.

## Data Availability Statement

The raw data supporting the conclusions of this article will be made available by the authors, without undue reservation.

## Ethics Statement

This study was approved by the Ethics Committee of the First Hospital of Jilin University (see **Supplementary Data Sheet 1**). Informed consent was obtained from all patients who participated in the study. All studies were conducted in accordance with the relevant regulations and guidelines.

## Author Contributions

JLW: methodology, software, data curation, and writing-original draft. JW: methodology and writing-original draft. HW and TZ: validation, formal analysis, and data curation. BW: software and resources. LM: formal analysis and writing-review and editing. LD: resources, writing-review and editing, project administration, and funding acquisition. XJ: conceptualization, resources, writing-review and editing, funding acquisition, and supervision. All authors contributed to the article and approved the submitted version.

## Funding

This work was supported by the National Key R&D Program of China (Grant number 2017YFC0112100); the Education Department Foundation of Jilin Province (Grant number JJKH20201036KJ); the Health and Family Planning Commission of Jilin Province Foundations (Grant number 2016Q034 and 2017J11); the Fundamental Research Funds for the Central Universities of Jilin University; and the Jilin Provincial Science and Technology Foundations (Grant number 20180414039GH and 20190201200JC).

## Conflict of Interest

The authors declare that the research was conducted in the absence of any commercial or financial relationships that could be construed as a potential conflict of interest.

## Abbreviations

BMI, Body mass index; HNC, Head and neck cancer; OM, Oral mucositis; PG-SGA, Patient-Generated-Subjective Global Assessment; QOL, Quality of life, RT: Radiotherapy; SD, Standard deviation.
