# Supplementary material for: A Bioadhesive Barrier-Forming Oral Liquid Gel Improved Oral Mucositis and Nutritional Status in Patients With Head and Neck Cancers Undergoing Radiotherapy: A Retrospective Single Center Study
Source: Front Oncol. 2021 Feb 22;11:617392. doi: 10.3389/fonc.2021.617392 (PMC7937867; doi:10.3389/fonc.2021.617392)
Supplement: Supplementary file 1 [file DataSheet_1.pdf]

吉林大学第一医院伦理委员会

审查意见

（2020 年）临审第（2020-400）号

**项目名称**	生物粘附性口腔凝胶对头颈部肿瘤放疗患者口腔黏膜炎和营养状况的改善：一项回顾性临床研究		
**项目来源**	吉林大学第一医院		
**专业名称**	放疗科	**主要研究者**	姜新
**审查类别**	初始审查	**审查方式**	快速审查
**主审委员**	牛俊奇；孙健		
**审查文件**	1. 临床研究方案（版本号：1.0；版本日期：2020 年 08 月 25 日）<br>2. 实验数据记录表格（版本号：1.0；版本日期：2020 年 08 月 25 日）<br>3. 主要研究者简历<br>4. 免知情同意申请（日期：2020 年 08 月 25 日）		
**审查意见**	同意		

根据 ICH-GCP、《涉及人的生物医学研究伦理审查办法》（2016）、《药物临床试验质量管理规范（2020）》、《医疗器械监督管理条例》（2017）、《医疗器械临床试验质量管理规范》、WMA《赫尔辛基宣言》和 CIOMS《人体生物医学研究国际道德指南》的伦理原则，经本伦理委员会审查，<u>同意</u> 该临床试验在本中心开展。

请遵循 GCP 原则、遵循伦理委员会审查同意的方案开展临床研究，保护受试者的健康与权力。

研究过程中若变更主要研究者，对临床试验方案、知情同意书、招募材料等的任何修改，请申请人提交修正案审查申请。

发生安全性事件，请申请人及时提交安全性报告。

请申请人按照伦理委员会规定的年度/定期跟踪审查频率，在截止日期前 1 个月提交研究进展报告；当出现任何可能显著影响试验进行、或增加受试者危险的情况时，请申请人及时向伦理委员会提交书面报告。

研究纳入了不符合纳入标准或符合排除标准的受试者，符合中止试验规定而为未让受试者退出研究，给予错误治疗或剂量，给予方案禁止的合并用药等没有遵从方案开展的情况；为了消除对受试者的紧急危害，研究者修改或者偏离试验方案或可能对受试者的权益/健康以及科学性造成不良影响等违背 GCP 原则的情况，请申办方/监察员/研究者提交违背方案报告。

申请人暂停/ 或提前终止临床研究，请及时提交暂停/终止研究报告。

完成临床研究，请申请人提交结题报告。

**年度/定期跟踪审查频率**	☐3 个月	☐6 个月	☑12 个月
**有效期**	本审查意见有效期为一年，须在截止日期前 1 个月递交研究进展报告，经伦理委员会审查同意后方可继续进行。		
**(副)主任签字**	[signature]		
**日期**	2020.8.26		

吉林大学第一医院伦理委员会

扫描全能王 创建

吉林大学第一医院伦理委员会快审主审委员签字表

一、吉林大学第一医院伦理委员会声明：

吉林大学第一医院伦理委员会的职责、人员组成、操作程序及记录符合 ICH-GCP、中国 GCP 以及国家相关规定。

二、审核时间：2020 年 08 月 26 日

三、吉林大学第一医院伦理委员会成员名单及快审主审委员签名

伦理委员会职务	姓　名	性别	工 作 单 位	职务/职称	专业	签字栏
主任委员	牛俊奇	男	吉林大学第一医院肝胆胰内科	主任/主任医师	传染病学<br>肝病学	[illegible]
副主任委员	姜　晶	女	吉林大学第一医院临床研究部	主任/教授	临床流行病学	
副主任委员	孙　健	男	吉林大学第一医院心血管内科	主任医师	心血管病学	[illegible]
委　员	崔　俐	女	吉林大学第一医院神经内科	主任/主任医师	神经病学	
委　员	李艳妍	女	吉林大学第一医院医学检验科	副主任/主任药师	药学	
委　员	刘　彬	男	吉林大学第一医院	副院长/主任医师	手足外科学	
委　员	刘　畅	女	吉林大学第一医院心理卫生科	主任/主任医师	临床心理学	
委　员	刘　丽	女	吉林大学第一医院药物临床试验机构办公室	主任/主任医师	儿科学	
委　员	吕美德	男	吉林大学第一医院肝胆胰内科	主任医师	传染病学<br>肝病学	
委　员	曲红梅	女	吉林大学哲学社会学院	副院长/教授	哲学	
委　员	续　薇	女	吉林大学第一医院检验科	主任医师	临床检验学	
委　员	严超英	女	吉林大学第一医院新生儿科	主任医师	儿科学	
委　员	姚　程	男	吉林大学第一医院肿瘤中心	主任医师	肿瘤学	
委　员	于德顺	男	常春律师事务所	律师	法律学	
委　员	俞　琼	女	吉林大学公共卫生学院	教授	流行病学	
委　员	张松灵	女	吉林大学第一医院科研部	主任/主任医师	妇产科学	
委　员	周　钢	男	吉林公正司法鉴定中心	主任	法律学	

吉林大学第一医院伦理委员会

※委员姓名按字母顺序排列，任职期限：2018 年 07 月 03 日至 2021 年 07 月 02 日

伦理委员会秘书：赵丽媛；郭迪

联系方式：0431-88782013

地址：吉林省长春市新民大街 1 号

扫描全能王 创建
